# New observation of perceptive mechanism behind the long-lasting change of people's community mobility: evidence from COVID-19 in China

**DOI:** 10.1038/s41598-023-32009-5

**Published:** 2023-03-30

**Authors:** Ziwen Ye, Yang Yu, Yuxin Liu, Chaosheng Zhang, Lei Huang

**Affiliations:** 1 State Key Laboratory of Pollution Control and Resource Reuse, School of the Environment, Nanjing University, Xianlin Campus, 163 Xianlin Avenue, Nanjing, 210023 China; 2 Nanjing University (Suzhou) High-Tech Institute, Suzhou, 215123 China; 3 Institute for Interdisciplinary Information Sciences, Tsinghua University, Beijing, 100084 China; 4 International Network for Environment and Health, School of Geography, Archaeology and Irish Studies, University of Galway, Galway, H91 CF50 Ireland

**Keywords:** Psychology and behaviour, Risk factors

## Abstract

COVID-19 pandemic provides an opportunity to investigate how a new and long-lasting threat affects public risk perception and social distancing behavior, which is important for pandemic risk management and recovery of the tertiary industry. We have found that the mechanism that perception decides behavior changes over time. At the beginning of the pandemic, risk directly shapes people’s willingness of going out. But under a persistent threat, perception no longer plays the direct role of shape people’s willingness. Instead, perception indirectly influences the willingness by shaping people’s judgment about the necessity of traveling. Switching from direct to indirect influence, perception’s effect is enlarged, which partially prevents people from returning to normal life even if the governmental ban is removed in a zero-COVID community.

## Introduction

Risk perception is a concept used to describe people's attitudes and subjective judgments about a series of risks^1,2^. Individuals' perceptions of objective issues inevitably affect their attitudes and behaviors toward objective issues. When an accident occurs, such as the Fukushima nuclear power accident^3,4^, the air pollution incident^5^, etc., risk perception is often incorporated into the management decision-making process. On the one hand, research on risk perception, which focuses on the changes in people's perception of risk before and after the accident, can promote risk communication and provide decision-making basis for risk management. On the other hand, it can provide guidance for formulating health intervention methods according to individual characteristics, so as to conduct risk management. Whether the risk perception affect individual choices by a static mechanism from the first day when the risk appears is a question that has not been sufficiently answered. It has been broadly discussed through which path the risk perception impacts the behaviors of individuals. However, most discussed risks such as the nuclear-power risk have been existing for a long time. It is reasonable to assume that the functional relation between the risk perception and individual choices is time-stable.

However, it could be different for some special risks. For instance, the risk of infection during the COVID-19 pandemic is a new but long-lasting threat. Our understanding of the risk innovates with the development of the pandemic. Unlike other accidents, the pandemic has the characteristics of long duration, global spread, large impact, and uncertainty^6–8^. The COVID-19 pandemic was discovered in Wuhan in December 2019, and then quickly spread globally. Although Wuhan was lifted from control on April 8th, 2020 when the local cases were cleared, the pandemic is still raging around the world. And some researchers have proposed that pandemic-level pandemic cycles may occur more frequently in the near future^9,10^. In China, strict isolation and control measures were taken whenever the epidemic spreads repeatedly, which has had a huge impact on the tertiary industry (including insufficient consumption power, reduced labor force in the economic sector, job losses, etc.)^11,12^. It has been demonstrated that the risk perception suppressed the willingness of individuals to travel during the pandemic^13–15^. Meanwhile, government policies increase the perceived risk and further reduce the frequency of public travel based on the protective action decision model^16,17^. It implies that risk perception can have a significant direct effect on behavioral intentions with a direct effect on behavior both during this COVID-19 outbreak^18,19^ and in the post-pandemic period^17,20^. However, the pathways through which the risk perception affects individual choices may change with time, which requires further investigation^21^. Similarly, it is also unclear whether government policies affect the risk perception by an innovative mechanism when a new threat occurs. Multiple studies have analyzed how government risk management shapes public risk perception^16^. However, few of them have examined whether the mechanism is changing over time. It is necessary to reveal the mechanism of development in the chain linking the government risk management and the choices of individuals. Thus, the 2020 COVID-19 pandemic is one of the first opportunities to investigate how an ongoing threat affects perceptions and behaviors, which is also extremely important for understanding the relationship between human perception and the spread of the pandemic. In this study, a retrospective questionnaire survey was conducted to analyze the functional relation between government risk management and public risk perception and their choices.

## Materials and methods

### Measurement

**Figure 1:**
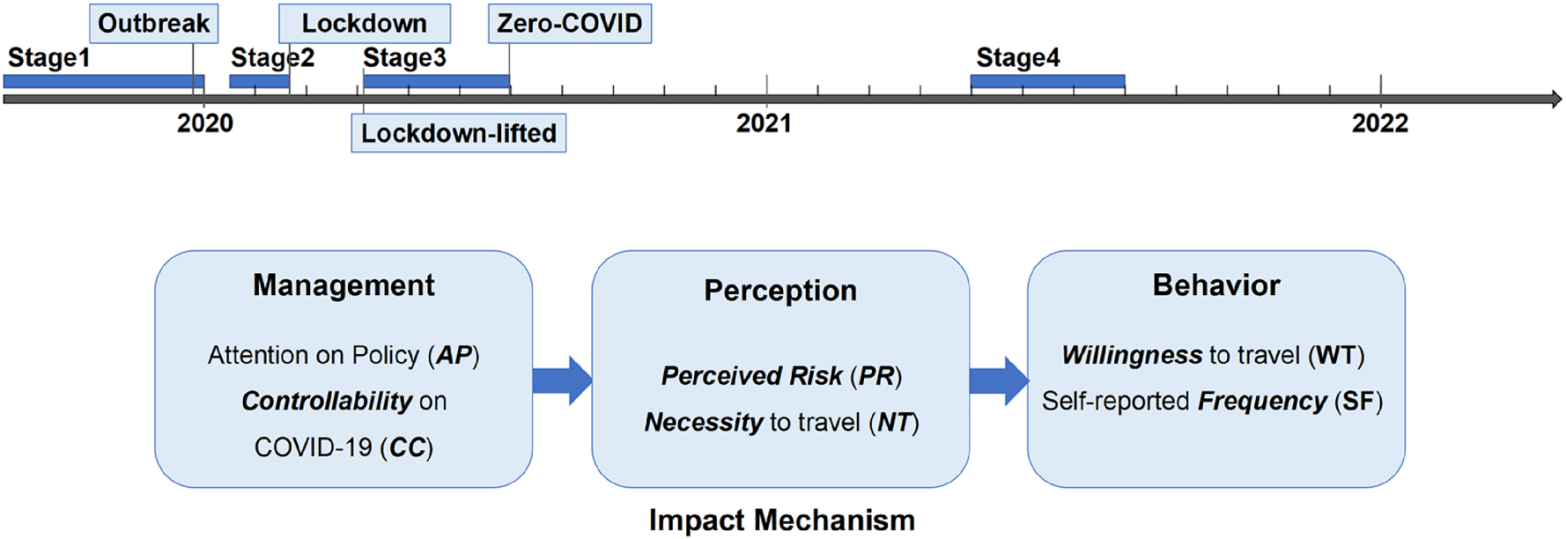
Graphic abstract of the design of the study.

We used a retrospective questionnaire to survey three aspects, including personal basic information, risk perception survey, travel willingness survey to six places during four stages, including Before-pandemic, Before-Lockdown (at the outbreak of pandemic), Lockdown-lifted (when the COVID cases were decreasing with the zero-COVID policy), and Post-pandemic (1 year after the Lockdown-lifted when the areas were without COVID cases) (Fig. 1). These four stages are representative of China before and after the epidemic, specifically number of cases in Figure S1. The risk perception survey and travel willingness survey were collected by a five-point Likert scale (Table 1, full questionnaire in Supplementary text 1).

**Table 1:** Risk perceptions and travel behavior variables investigated at various stages.

Variables	Questions	Stages
Management		
Attention on policy	Are you concerned about the pandemic prevention policy related to the following places?	Stage2–4
Controllability on COVID-19	Do you think the pandemic situation in the following places can be controlled through relevant pandemic prevention and control policies?	Stage2–4
Risk perceptions		
Perceived Risk	How risky do you think you are at contracting COVID-19?	Stage2–4
Necessity to travel	How necessary did you think it is to go to the following places?	All
	Why did you think it is very necessary to go to this place?	All
Travel behavior		
Willingness to travel	Were you willing to go to the following places?	Stage2–4
Self-reported Frequency	How often do you think you travel to the following places?	Stage1
	How do you think your frequency of travel has changed compared to 2019?	Stage2–4
	How many times a week did you go to the following places?	All
Outdoor Behavior	Where was the first place you went after the community was unblocked?	Stage3

### Data and procedure

We commissioned ‘Netease Dingwei’ to distribute questionnaires to adults living in Wuhan urban areas online. We got the informed consent of the respondents at the beginning of each questionnaire and promised that they could freely decide whether to continue. We first conducted a preliminary survey to collect 50 questionnaires in Wuhan in July 2021. According to the results of data cleaning and data analysis, we adjusted the questionnaire structure. Then, from July to August 2021, the questionnaires will be officially distributed by random stratified sampling online. A total of 450 valid questionnaires were recovered which is consistent with the minimum sample size for stratified random sampling (Equation in Supplementary text 2). The sample was well controlled for population proportion stratification which is consistent with the actual ratio of Wuhan (see Supplementary Table S1 online). The Ethics Committee of School of the Environment, Nanjing University approved the protocol of this study. We confirmed that all methods were carried out in accordance with relevant guidelines and regulations as well as performed in accordance with the Declaration of Helsinki.

### Analytical approaches

**Figure 2:**
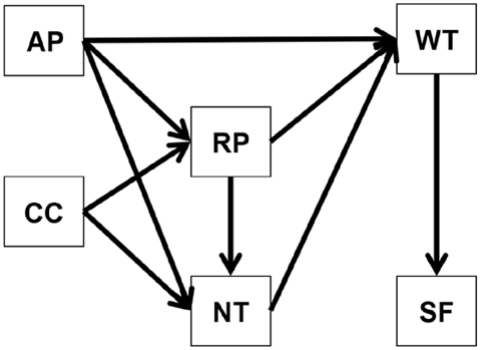
The SEM of this study.

First, the stage changes of perception and willingness were analyzed. And Spearman correlation was used to analyze the correlation of factors. Then, considering the random effects of multiple sampling, we used GLMM analysis (R package^22,23^) to estimate the factors affecting willingness and identify the main influencing factors (see variable allocation in Supplementary Table S2). According to the results of influencing factors, SEM analysis was further adopted to study and analyze the changes of action mechanism before and after the accident to verify the hypothesis (Fig. 2). Finally, regression analysis was performed to estimate the loss of frequency for different impact paths. The analysis was conducted by IBM SPSS version 22.0, AMOS version 22.0, and R version 4.1.1.

## Results and discussion

### The necessity and frequency of travel have not returned to pre-pandemic levels

**Figure 3:**
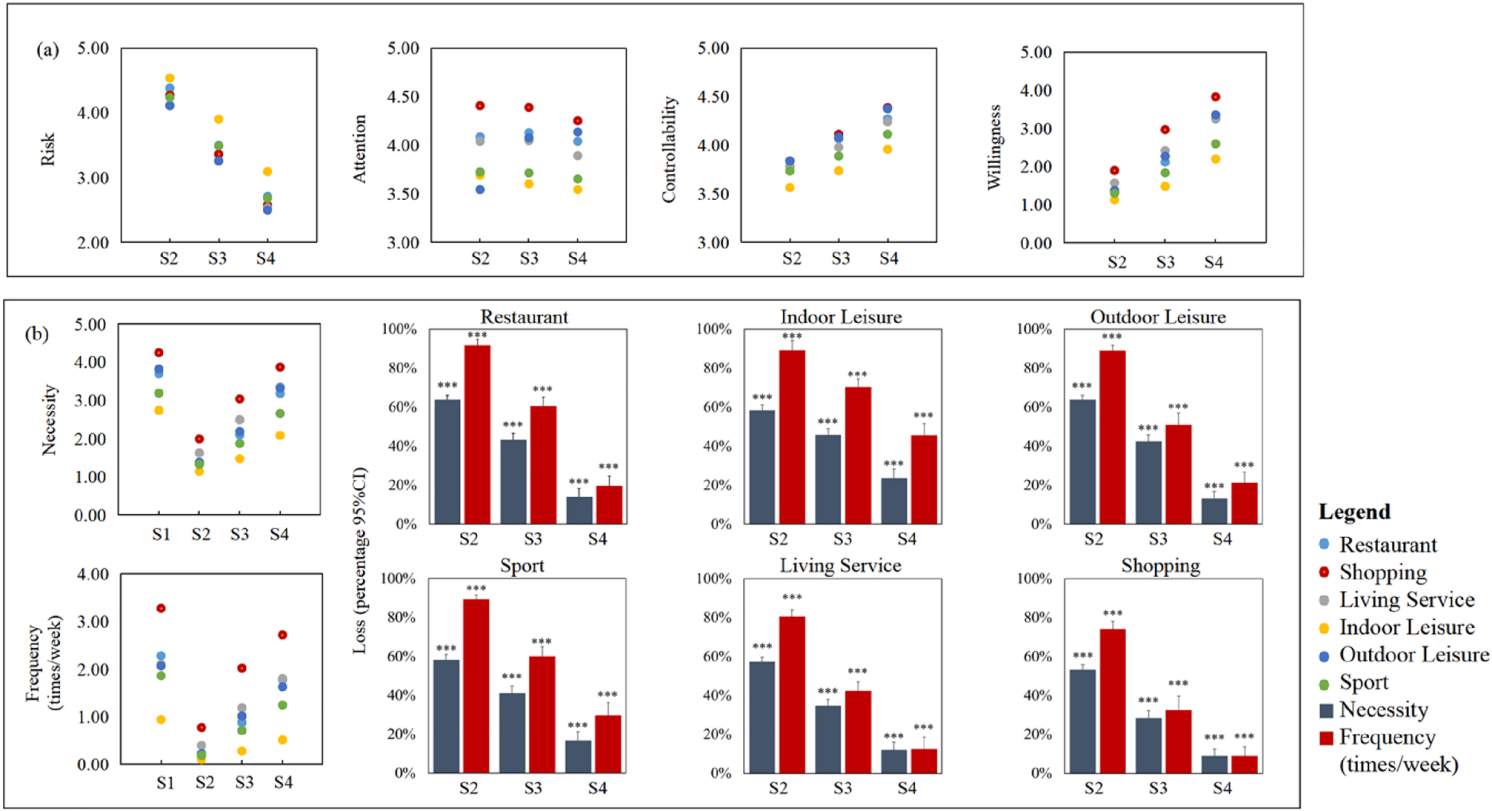
Changes over four stages. (**a**) Changes of perception. (**b**) Loss of necessity and frequency (times/week) during stage2 to stage4. ***p < 0.001, the comparison of stage n and stage1 (n = 2, 3, 4).

Figure 3a shows the changes of five perception dimensions and self-reported frequency of six places during the four stages. After the outbreak of pandemic, travel in stage2 was restricted by policies, thus the self-reported travel frequency decreased has dropped significantly, which is consistent with many studies. With the control of the pandemic from stage2 to stage4, the residents' perceived risk declined significantly, while the controllability and the willingness of residents increased. And the higher the perceived risk, the lower the willingness, necessity and self-reported travel frequency (see Supplementary Tables S3–S8 online).

The travel frequency increased from stage2 to stage4 with the liberalization of policy. The necessity of six places declined in stage2, and increased from stage2 to stage4 as well. However, in stage4, when confirmed cases had been cleared and travel was not restricted by policies, the necessity and frequency was also lower than that in stage1. The loss of public necessity and frequency from stage2 to stage4 compared to stage1 is shown in Fig. 3b. Differences in stage4 remained significant, which mean that the change of necessity and frequency are asymmetric. Although the pandemic has recovered, the necessity and frequency of travel have not.

In addition, the difference in perception among six places shows a certain law. Indoor leisure places were considered as the highest risky places, with the lowest controllability, willingness, necessity and frequency. And shopping places were the most necessary places.

This is similar to many previous studies. Studies have divided social distancing behavior in pandemics into policy-induced behavior and voluntary behavior^24^. And people still maintain voluntary social distancing behaviors after the epidemic disappears, such as reducing the frequency of travel^25^, choosing less risky locations^26^, choosing safer travel transportation^27^, etc. And risk perception will affect people to comply with COVID-19 social distance restrictions^28^.

### Necessity is the main factor directly influencing travel willingness under a durable threat

We used the GLMM model to explore the contributing factors of changes in willingness. We found that the willingness to travel to the six places changed obviously in different stages. As shown in Table 2, necessity is the main factor affecting willingness (value > 25%, p < 0.001). Risk perception is also an important factor affecting travel willingness to the six places. Except for shopping places, the perceived risk has a negative impact on willingness significantly. Attention on policy has a positive impact on willingness. Controllability affected the public's willingness to go to living service places.

**Table 2:** GLMM model of willingness.

	A—Restaurant			B—Outdoor leisure		
	Value	95% CI	p	Value	95% CI	P
(Intercept)	− 0.436	− 4.789 to 3.917	< 0.001	− 0.387	− 4.614 to 3.839	< 0.001
Necessity	0.270	− 0.094 to 0.633	< 0.001	0.285	− 0.070 to 0.639	< 0.001
Risk	− 0.039	− 0.377 to 0.299	< 0.001	− 0.039	− 0.376 to 0.298	< 0.001
Attention	0.035	− 0.373 to 0.444	< 0.001	0.021	− 0.368 to 0.409	0.027
Controllability	0.011	− 0.415 to 0.436	0.295	0.010	− 0.403 to 0.424	0.297
Gender	− 0.029	− 0.795 to 0.738	0.123	0.004	− 0.730 to 0.737	0.838
Age	− 0.021	− 0.277 to 0.235	0.001	− 0.013	− 0.260 to 0.235	0.033
Education	0.017	− 1.104 to 1.138	0.521	− 0.023	− 1.078 to 1.032	0.366
Stage	0.166	− 0.706 to 1.038	< 0.001	0.163	− 0.721 to 1.048	< 0.001
Population	0.042	− 0.762 to 0.845	0.032	0.052	− 0.723 to 0.827	0.005
Income	0.009	− 0.541 to 0.559	0.499	0.008	− 0.520 to 0.536	0.547

	C—Intdoor leisure			D—Sport		
	Value	95% CI	p	Value	95% CI	p
(Intercept)	− 0.421	− 5.425 to 4.582	< 0.001	− 0.414	− 5.042 to 4.215	< 0.001
Necessity	0.311	− 0.127 to 0.748	< 0.001	0.312	− 0.067 to 0.691	< 0.001
Risk	− 0.020	− 0.383 to 0.343	0.022	− 0.031	− 0.375 to 0.313	< 0.001
Attention	0.021	− 0.350 to 0.393	0.017	0.051	− 0.304 to 0.406	< 0.001
Controllability	0.016	− 0.385 to 0.417	0.098	0.003	− 0.391 to 0.396	0.782
Gender	0.001	− 0.977 to 0.979	0.973	− 0.029	− 0.906 to 0.847	0.166
Age	− 0.025	− 0.350 to 0.301	0.002	− 0.012	− 0.304 to 0.280	0.089
Education	− 0.014	− 1.411 to 1.383	0.672	− 0.004	− 1.279 to 1.270	0.884
Stage	0.097	− 0.787 to 0.981	< 0.001	0.079	− 0.764 to 0.922	< 0.001
Population	0.032	− 0.991 to 1.056	0.189	0.028	− 0.894 to 0.950	0.212
Income	< 0.001	− 0.702 to 0.702	0.995	0.028	− 0.607 to 0.663	0.064

	E—Living service			F—Shopping		
	Value	95% CI	p	Value	95% CI	p
(Intercept)	− 0.309	− 4.233 to 3.615	< 0.001	− 0.423	− 3.952 to 3.105	< 0.001
Necessity	0.270	− 0.077 to 0.618	< 0.001	0.254	− 0.043 to 0.551	< 0.001
Risk	− 0.026	− 0.350 to 0.297	0.001	− 0.010	− 0.288 to 0.268	0.142
Attention	0.032	− 0.337 to 0.401	< 0.001	0.026	− 0.340 to 0.393	0.003
Controllability	0.025	− 0.370 to 0.421	0.008	0.015	− 0.331 to 0.360	0.076
Gender	− 0.002	− 0.686 to 0.681	0.884	0.001	− 0.573 to 0.574	0.965
Age	− 0.016	− 0.242 to 0.210	0.004	0.001	− 0.191 to 0.193	0.791
Education	< 0.001	− 0.972 to 0.972	0.992	0.037	− 0.804 to 0.878	0.066
Stage	0.122	− 0.706 to 0.950	< 0.001	0.145	− 0.569 to 0.858	< 0.001
Population	− 0.001	− 0.715 to 0.713	0.955	0.010	− 0.595 to 0.616	0.474
Income	− 0.001	− 0.488 to 0.487	0.940	0.010	− 0.400 to 0.420	0.325

The demographic factors affecting the willingness to travel varied a lot. Age significantly affected residents’ willingness to go to restaurants, leisure places (indoor and outdoor), and living service places. The family population affected the willingness to restaurants and outdoor leisure places. The willingness to leisure places (indoor and outdoor) and living places were also affected by the vaccine. Sport places were less affected by demographic factors. The influence of demographic factors on perception found in previous studies varies as well^29,30^. However, many studies also found that demographics factors have no significant effect on travel intention^31^. Therefore, based on the instability of demographic factors, we did not consider the influence of population factors in the following SEM analysis to focus on discussion of the relationship between perceptions and behaviors, which does not mean that the impact of demographic factors is not important.

### New observation of the role of risk perception in linking government risk management and public behaviors

**Figure 4:**
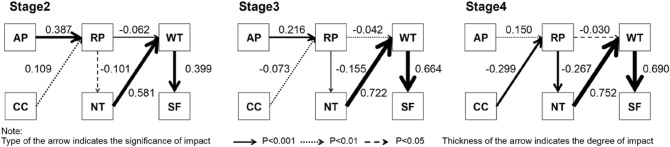
Path analysis of impact paths on self-reported frequency to travel during stage2 to stage4. Confirmatory factor analysis and Goodness-of-fit statistics in Table S9–12.

Figure 4 shows that the impact of controllability and attention on necessity has changed from being largely ignored to being appropriately considered. This creates the effect of these two factors, amplifying the effect on the frequency of visits not only by Attention but also by necessity. This indicates that the development of the functional relationship between government risk management and public risk perception. Public attention on government policy has a consistent and significant positive influence on perceived risk but to a lesser extent when the pandemic is under control. While the impact of public belief in the controllability of COVID-19 on perceived risk has fundamentally changed. With the implementation of the zero-COVID policy, the influence of belief in controllability of COVID-19 on perceived risk has changed from positive influences to negative influences.

It also demonstrated the change in the pathways through which public risk perception influences their behavior. Although risk perception always plays a decisive role when individuals make mobility decisions, the influencing pathways are different between the pandemic outbreak and the zero-COVID environment. However, when the environment is zero-COVID, the influence of perceived risk on willingness has changed from direct influence to indirect influence through necessity. The impact of risk perception on the necessity and the impact of necessity on willingness are both strong. Moreover, we found that one unit of risk perception has a stronger blocking effect on the willingness in the zero-COVID environment than during the outbreak.

### New observation of risk perception’s pathway: a candidate theory for the long-lasting voluntary social distancing

The correlation analysis of perceived risk, necessity, and willingness changed from stage2 to stage4 (Fig. 4). The decision mechanism of the frequency of visits has changed. Necessity affected willingness during all three stages, while, from stage2 to stage4, the significance of risk perception influence on willingness decreases, and the significance of risk perception influence on necessity increases. Thus, the influence of risk perception on the willingness to visit has changed from direct influence to indirect influence through necessity.

Effects of perceived risk have changed after the pandemic resumed. In stage2 and stage3, the impact of risk on willingness was significant. The willingness depended more on the perceived risk. However, in stage4, though the effect remained, it was lower than that in the pandemic. Compared with before the pandemic, the relationship between the necessity and risk varies by 2–4 times. Willingness was not directly affected by risk, but by necessity which had a significant impact on willingness during all three stages. Compared with the stage before the pandemic, necessity after the pandemic is more determined by risk. The results showed that the impact of perceived risk on necessity was hysteretical, and asymmetrical. One of the reasons for this is that perceived risk has a limited impact on necessity. If the magnitude and mechanism of risk's impact on perceived risk remain unchanged, people's willingness to visit will not bounce back even if the risk is reduced to its previous position. This asymmetry determines that although the perceived risk has recovered, while the frequency has not fully recovered. Thus, even though the perceived risk of people in the fourth stage is reduced, the effect on frequency is amplified by necessity.

It provides a novel explanation for the long-lasting voluntary social distance. The statistics about community mobility since the start of the coronavirus pandemic revealed permanent and profound changes in the psychological dynamics behind the behavior of individuals. Evidence indicates that the number of visitors to business sites and workplaces has not been back to the pre-pandemic level although the government remove the mandatory social-distancing orders or even in the areas without COVID cases. Literature has confirmed that it is the risk perception deciding the behavior under the threat of a possible accident^15^. However, it is still a mystery what is the motivation preventing people from visiting business sites and working places areas without COVID cases. The theory about the change of the role of risk perception provides a candidate theory for long-lasting voluntary social distance.

**Figure 5:**
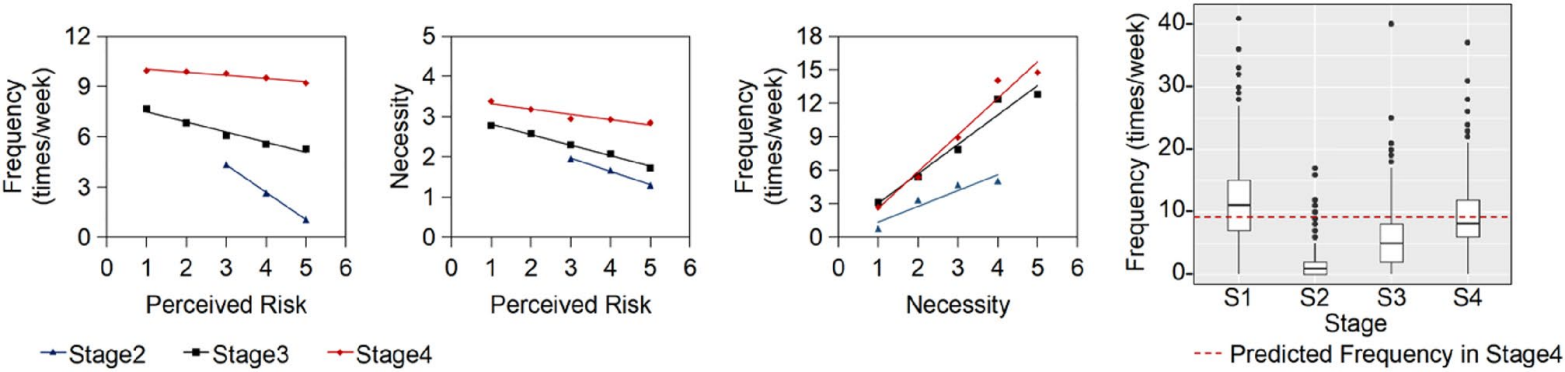
Linear relationship between perceived risk, necessity, and frequency.

Our discovery manifests that it is the change in the mechanism by which public risk perception determines their willingness to travel, resulting in the travel frequency not recovering in an environment without COVID cases. Because the risk perception’s role changed from direct impactor to indirect impactor, the impact of risk perception was even multiplied. In contrast, if risk perception has always directly affected travel behavior, the change in travel behavior with perceived risk should be symmetrical. For instance, we compare the frequency of stage3 and stage4 in two scenarios: the real-world scenario when the impact of risk perception changes from the direct to indirect, and the counter-factual scenario when the risk perception always directly determines the will according to the mechanism in stage. (Fig. 5) In the counterfactual scenario, the one-unit increase of the perceived risk led to a 0.62-times decrease in the frequency per week in stage3. In the real-world scenario, the frequency decreased by 0.69 times per week, which was 13% more than that in the counterfactual scenario. In stage4, the difference between the counterfactual scenario and the real-world scenario was even enlarged. A one-unit increase in perceived risk directly led to a decrease of necessity by 0.13 units, which in turn led to a decrease of frequency by 0.43 times per week in the real-world scenario. In the counterfactual scenario, the one-unit increase of the perceived risk would lead to an only 0.19-times decrease in the frequency per week in stage4. Per-unit risk-perception increase caused a 2.3-times more traveling decrease due to the change of the mechanism that risk perception influences the frequency.

Therefore, by switching from direct influencing to indirect affecting, the effect of risk perception of discouraging traveling was amplified. A moderate risk perception will cause a significant decrease in traveling when zero-COVID than during the outbreak of the pandemic, which causes voluntary social distancing.

## Conclusion

In summary, it can be seen that when people perceive that the risk is getting smaller, the frequency of the rebound visit is not responded, mainly because of the structural change in the formation mechanism of people's judgment of “whether it is necessary to go to a place”. When there is no pandemic, or in the early stage of the pandemic, people's judgment on the necessity to go to a place has nothing to do with the risk of the pandemic. However, with the development of the pandemic, factors related to risk and risk management are included as a basis for judgment on the necessity to travel. Due to the fundamental structural changes in the frequency impact mechanism of perception of risk and risk control before and after the pandemic, from basically not affecting people's behavior through the influence of necessity, to affecting people through the influence of necessity behavior, resulting in the phenomenon that people cannot go back. Thus, this is the perception-frequency relationship, the mechanism by which the asymmetry (decoupling) is restored.

Our results reveal a novel dimension of the study of risk perception beyond the rational and emotional motivations. We suggest the change in the mechanism of risk perception influencing the willingness is a novel direction for the study of human risk perception and the associated impacts. In-depth studies are still required on why the risk-based decision-making mechanism has changed. The majority of the current discussion about risk perception mainly focuses on either rational tradeoffs or emotional behavior such as information nudges^32^. However, the above analysis demonstrates that the mechanism of risk perception deciding the people’s mobility was innovative during the pandemic, and it contributed to the long-lasting voluntary social distancing. Our discoveries stimulate future studies on the change of the mechanism that the risk perception determines the behavior. For instance, it is necessary to examine whether the change was a side effect of the accident on psychology. How does the change interact with the digital transition of society? All these issues about the new observation of the mechanism that risk perception determines the behavior is necessary for us to deepen our insights on the psychological effect of the risk, and the associated social consequences.

Limitation also exists in this study. Due to the length limitation, the questionnaire in this study used only one question for each perception dimension. And there are indeed a very large number of factors that can influence risk perception and behavior, we did not include some other factors, such as psychological constructs^33^, in our study analysis due to the limitations of our study design. Further research can be designed and carried out in the future. In addition, our research data are mostly subjective, and the sample size is more biased toward people with high education, and subsequent research can be combined with objective location data such as GPS or POI for site experiment verification analysis.

## Supplementary Information

Supplementary Information.

## Data Availability

All relevant data are within the manuscript and its Supporting Information files. The data that support the findings of this study are available upon request from the corresponding author, by sending email to Lei Huang; Email: huanglei@nju.edu.cn.
