# Supplementary material for: New observation of perceptive mechanism behind the long-lasting change of people's community mobility: evidence from COVID-19 in China
Source: Sci Rep. 2023 Mar 30;13:5165. doi: 10.1038/s41598-023-32009-5 (PMC10060925; doi:10.1038/s41598-023-32009-5)

**Supplementary Information for:**

New Observation of Perceptive Mechanism Behind the Long-lasting Change of People's Community Mobility: Evidence from COVID-19 in China

Ziwen Ye1,2, Yang Yu3, *, Chaosheng Zhang4, Lei Huang1,2, **

1 State Key Laboratory of Pollution Control & Resource Reuse, School of the Environment, Nanjing University, Xianlin Campus, 163 Xianlin Avenue, Nanjing, 210023, China

2 Nanjing University (Suzhou) High-Tech Institute, Suzhou 215123, China

3 Institute for Interdisciplinary Information Sciences, Tsinghua University, Beijing, 100084, China

4 International Network for Environment and Health, School of Geography, Archaeology and Irish Studies, University of Galway, Galway, H91 CF50, Ireland

*Correspondence author: Yang Yu; Email: yangyu1@tsinghua.edu.cn

**Corresponding author: Lei Huang; Email: huanglei@nju.edu.cn

**This PDF file includes:**

Supplementary text 1

Supplementary text 2

Table S1 to S12

Figure S1

**Supplementary text 1 Questionnaire**

**Questionnaire on risk perception of six places during different stage in Wuhan, China**

This survey is a cooperative research project between Tsinghua University and Nanjing University. The information collected in the survey is for scientific research purposes only and does not involve personal privacy. We will keep the survey data strictly confidential. The filling time is about 8-10 minutes, thank you for your precious time!

Restaurant

Outdoor Leisure Places: parks, amusement parks, national attractions, etc.

Indoor Leisure Places: KTV, bars, clubs, etc.

Sport Places: gym, etc.

Living Service Places: barber shop, postal point, etc.

Shopping Places: markets, malls, etc.

**Part 1**

Q1. What is your resident city?

__________

Q2. Your gender?

○male ○female

Q3. Your age?

__________

Q4. Your education level?

○Elementary school ○Junior high school ○High school/vocational ○University/vocational school ○Graduate and above

Q5. Your occupation?

○Civil servants ○Personnel of public institutions ○Personnel of enterprises ○Self-employed ○Workers ○Teachers ○Medical staff ○Researcher ○Military ○Farmer ○Housewife ○Student ○Freelance ○Retired ○Other

Q6. Monthly household income (yuan) :

○＜1,000 ○1,000-2,000 ○2,000-5,000 ○5,000-10,000 ○100,000-20,000 ○＞2 million

Q7. How many people are there in your family?

________

Q8. What area do you live in?

○Urban ○Rural

Q9. Which of the following places is not a landmark in Wuhan?

○
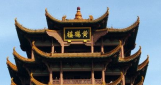
○
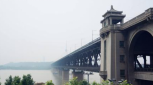
○
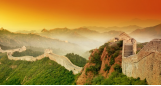
○
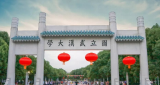


**Part 2**

**Stage 1 Before the epidemic**

| Read the following scenarios and answer the questions. Please read the options carefully.  **Period**: 2019  **Epidemic**: Before the epidemic |
| --- |

1、In 2019, how often do you think you travel to the following places?

|  | Almost None | Mild | Neutral | Often | Always |
| --- | --- | --- | --- | --- | --- |
| Restaurant |  |  |  |  |  |
| Outdoor Leisure Places |  |  |  |  |  |
| Indoor Leisure Places |  |  |  |  |  |
| Sport Places |  |  |  |  |  |
| Living Service Places |  |  |  |  |  |
| Shopping Places |  |  |  |  |  |

2. In 2019, how necessity did you think it is to go to the following places?

|  | Almost None | Mild | Neutral | Quite | Strong |
| --- | --- | --- | --- | --- | --- |
| Restaurant |  |  |  |  |  |
| Outdoor Leisure Places |  |  |  |  |  |
| Indoor Leisure Places |  |  |  |  |  |
| Sport Places |  |  |  |  |  |
| Living Service Places |  |  |  |  |  |
| Shopping Places |  |  |  |  |  |

2.1 In 2019, why did you think it is very necessary to go to this place?

|  | Totally disagree | Relatively disagree | Neutral | Relatively agree | Totally agree |
| --- | --- | --- | --- | --- | --- |
| For living |  |  |  |  |  |
| For social relations |  |  |  |  |  |
| For work |  |  |  |  |  |
| For mental health |  |  |  |  |  |

3、In 2019, how many times a week did you go to the following places?

Restaurant times per week

Outdoor Leisure Places times per week

Indoor Leisure Places times per week

Sport Places times per week

Living Service Places times per week

Shopping Places times per week

**Stage 2 Before the community is blocked**

| Read the following scenarios and answer the questions. Please read the options carefully.  **Period**: January 23, 2020-February 14, 2020  January 25, 2020 Spring Festival, February 8, 2020 Lantern Festival  **Policy**:   \| January 23, 2020 \| Wuhan began to close down, and all public transportation was suspended \| \| --- \| --- \| \| January 24, 2020 \| Hubei Province launched the first-level response to major public health emergencies \| \| January 30, 2020 \| The World Health Organization declared the novel coronavirus outbreak an "International Public Health Emergency" \| \| February 4, 2020 \| Wuhan Huoshenshan Hospital was completed and put into use \| \| February 14, 2020 \| Wuhan requires all residential communities to be closed for management \|   **Epidemic**:  The number of confirmed cases of Wuhan Covid-19 continues to grow.  As of 24:00 on February 14, 2020, Hubei Province has reported a total of 54,406 cases of new coronary pneumonia, of which 37,914 were in Wuhan. |
| --- | --- | --- | --- | --- | --- | --- | --- | --- | --- | --- |

1、From January 23, 2020 to February 14, 2020, how do you think your frequency of travel has changed compared to 2019？

|  | Much Lower | Lower | Similar | Higher | Much higher |
| --- | --- | --- | --- | --- | --- |
| Restaurant |  |  |  |  |  |
| Outdoor Leisure Places |  |  |  |  |  |
| Indoor Leisure Places |  |  |  |  |  |
| Sport Places |  |  |  |  |  |
| Living Service Places |  |  |  |  |  |
| Shopping Places |  |  |  |  |  |

2、From January 23, 2020 to February 14, 2020, were you willing to go to the following places？

|  | Almost None | Mild | Neutral | Quite | Strong |
| --- | --- | --- | --- | --- | --- |
| Restaurant |  |  |  |  |  |
| Outdoor Leisure Places |  |  |  |  |  |
| Indoor Leisure Places |  |  |  |  |  |
| Sport Places |  |  |  |  |  |
| Living Service Places |  |  |  |  |  |
| Shopping Places |  |  |  |  |  |

3、From January 23, 2020 to February 14, 2020, how necessity did you think it is to go to the following places？

|  | Almost None | Mild | Neutral | Quite | Strong |
| --- | --- | --- | --- | --- | --- |
| Restaurant |  |  |  |  |  |
| Outdoor Leisure Places |  |  |  |  |  |
| Indoor Leisure Places |  |  |  |  |  |
| Sport Places |  |  |  |  |  |
| Living Service Places |  |  |  |  |  |
| Shopping Places |  |  |  |  |  |

3.1From January 23, 2020 to February 14, 2020, Why did you think it is very necessary to go to this place？

|  | Totally disagree | Relatively disagree | Neutral | Relatively agree | Totally agree |
| --- | --- | --- | --- | --- | --- |
| For living |  |  |  |  |  |
| For social relations |  |  |  |  |  |
| For work |  |  |  |  |  |
| For mental health |  |  |  |  |  |

4、From January 23, 2020 to February 14, 2020, How risky do you think you are of contracting COVID-19？

|  | Almost None | Mild | Neutral | Quite | Strong |
| --- | --- | --- | --- | --- | --- |
| Restaurant |  |  |  |  |  |
| Outdoor Leisure Places |  |  |  |  |  |
| Indoor Leisure Places |  |  |  |  |  |
| Sport Places |  |  |  |  |  |
| Living Service Places |  |  |  |  |  |
| Shopping Places |  |  |  |  |  |

5、From January 23, 2020 to February 14, 2020, Are you concerned about the epidemic prevention policy related to the following places？

|  | Almost None | Mild | Neutral | Quite | Strong |
| --- | --- | --- | --- | --- | --- |
| Restaurant |  |  |  |  |  |
| Outdoor Leisure Places |  |  |  |  |  |
| Indoor Leisure Places |  |  |  |  |  |
| Sport Places |  |  |  |  |  |
| Living Service Places |  |  |  |  |  |
| Shopping Places |  |  |  |  |  |

6、From January 23, 2020 to February 14, 2020, Do you think the epidemic situation in the following places can be controlled through relevant epidemic prevention and control policies?

|  | Almost None | Mild | Neutral | Quite | Strong |
| --- | --- | --- | --- | --- | --- |
| Restaurant |  |  |  |  |  |
| Outdoor Leisure Places |  |  |  |  |  |
| Indoor Leisure Places |  |  |  |  |  |
| Sport Places |  |  |  |  |  |
| Living Service Places |  |  |  |  |  |
| Shopping Places |  |  |  |  |  |

7、From January 23, 2020 to February 14, 2020, how many times a week did you go to the following places?

Restaurant times per week

Outdoor Leisure Places times per week

Indoor Leisure Places times per week

Sport Places times per week

Living Service Places times per week

Shopping Places times per week

**Stage 3 After unblocking**

| Read the following scenarios and answer the questions. Please read the options carefully.  **Period**：From April 8, 2020 to June 30, 2020  **Policy**：   \| April 8, 2020 \| Wuhan City is fully unblocked, and trains and flights from Wuhan City to other provinces are resumed \| \| --- \| --- \| \| May 2, 2020 \| Adjusted the emergency response level of public health emergencies in Hubei Province from Level 1 to Level 2 \| \| May 6, 2020 \| High school starts \| \| June 8, 2020 \| Graduates of colleges and universities and students with scientific research tasks return to school \| \| June 13, 2020 \| The emergency response level of Hubei Province's new crown pneumonia epidemic prevention and control has been adjusted from the second-level response to public health emergencies to the third-level response \|   **Epidemic**:  From April 18, 2020, all cities (counties, districts) in the province have been reduced to low risk.  On April 18, 2020, the death cases in Wuhan were revised. As of 24:00 on April 16, Wuhan had a total of 50,333 confirmed cases (an increase of 325), a total of 3,869 deaths (an increase of 1,290), and a total of 46,335 discharged cases. cases (965 cases were reduced).  On April 24, 2020, severe cases in Wuhan were cleared.  As of 24:00 on April 30, 2020, there are 0 confirmed cases and 0 suspected cases in Hubei Province. There have been no new cases for 27 consecutive days in the province, and there have been no new cases for 57 consecutive days in the province except Wuhan. |
| --- | --- | --- | --- | --- | --- | --- | --- | --- | --- | --- |

1、From April 8, 2020 to June 30, 2020, How do you think your frequency of travel to the following places has changed compared to 2019？

|  | Much Lower | Lower | Similar | Higher | Much higher |
| --- | --- | --- | --- | --- | --- |
| Restaurant |  |  |  |  |  |
| Outdoor Leisure Places |  |  |  |  |  |
| Indoor Leisure Places |  |  |  |  |  |
| Sport Places |  |  |  |  |  |
| Living Service Places |  |  |  |  |  |
| Shopping Places |  |  |  |  |  |

2、From April 8, 2020 to June 30, 2020, Are you willing to go to the following places？

|  | Almost None | Mild | Neutral | Quite | Strong |
| --- | --- | --- | --- | --- | --- |
| Restaurant |  |  |  |  |  |
| Outdoor Leisure Places |  |  |  |  |  |
| Indoor Leisure Places |  |  |  |  |  |
| Sport Places |  |  |  |  |  |
| Living Service Places |  |  |  |  |  |
| Shopping Places |  |  |  |  |  |

3、From April 8, 2020 to June 30, 2020, How necessity did you think it is to go to the following places？

|  | Almost None | Mild | Neutral | Quite | Strong |
| --- | --- | --- | --- | --- | --- |
| Restaurant |  |  |  |  |  |
| Outdoor Leisure Places |  |  |  |  |  |
| Indoor Leisure Places |  |  |  |  |  |
| Sport Places |  |  |  |  |  |
| Living Service Places |  |  |  |  |  |
| Shopping Places |  |  |  |  |  |

3.1From April 8, 2020 to June 30, 2020, Why did you think it is very necessary to go to this place？

|  | Totally disagree | Relatively disagree | Neutral | Relatively agree | Totally agree |
| --- | --- | --- | --- | --- | --- |
| For living |  |  |  |  |  |
| For social relations |  |  |  |  |  |
| For work |  |  |  |  |  |
| For mental health |  |  |  |  |  |

4、From April 8, 2020 to June 30, 2020, How risky do you think you are of contracting COVID-19？

|  | Almost None | Mild | Neutral | Quite | Strong |
| --- | --- | --- | --- | --- | --- |
| Restaurant |  |  |  |  |  |
| Outdoor Leisure Places |  |  |  |  |  |
| Indoor Leisure Places |  |  |  |  |  |
| Sport Places |  |  |  |  |  |
| Living Service Places |  |  |  |  |  |
| Shopping Places |  |  |  |  |  |

5、From April 8, 2020 to June 30, 2020, Are you concerned about the epidemic prevention policy related to the following places？

|  | Almost None | Mild | Neutral | Quite | Strong |
| --- | --- | --- | --- | --- | --- |
| Restaurant |  |  |  |  |  |
| Outdoor Leisure Places |  |  |  |  |  |
| Indoor Leisure Places |  |  |  |  |  |
| Sport Places |  |  |  |  |  |
| Living Service Places |  |  |  |  |  |
| Shopping Places |  |  |  |  |  |

6、From April 8, 2020 to June 30, 2020, Do you think the epidemic situation in the following places can be controlled through relevant epidemic prevention and control policies?？

|  | Almost None | Mild | Neutral | Quite | Strong |
| --- | --- | --- | --- | --- | --- |
| Restaurant |  |  |  |  |  |
| Outdoor Leisure Places |  |  |  |  |  |
| Indoor Leisure Places |  |  |  |  |  |
| Sport Places |  |  |  |  |  |
| Living Service Places |  |  |  |  |  |
| Shopping Places |  |  |  |  |  |

7、When was your community unblocked?？

○Early March ○Late March ○Early April ○Late April ○After May

8、Where was the first place you went after the community was unblocked？

○Restaurant ○Outdoor Leisure Places ○Indoor Leisure Places

○Sport Places ○Living Service Places ○Shopping Places

9、From April 8, 2020 to June 30, 2020, how many times a week did you go to the following places?

Restaurant times per week

Outdoor Leisure Places times per week

Indoor Leisure Places times per week

Sport Places times per week

Living Service Places times per week

Shopping Places times per week

**Stage 4 One year after unblocking**

| Read the following scenarios and answer the questions. Please read the options carefully.  **Period**：From May 1, 2021 to July 31, 2021  **Epidemic**：  At 10:00 on May 18, 2021, there were 0 new confirmed cases of new coronary pneumonia, 0 new suspected cases, and 0 new deaths in the province. There are 6 confirmed cases in the province (all imported from abroad).  On May 16, 2021, the city and district CDCs sampled 2,793 environmental samples, 108 animal food samples, and 28 employees. The nucleic acid test results for the new coronavirus were all negative. In addition, in the special monitoring of cold chain food, the city monitored 2 pieces of frozen food, 23 food packagings and 219 environmental samples in 26 freezers, and the nucleic acid test results of the new coronavirus were all negative.  As of 5:00 pm on May 17, 2021, Wuhan City has reported more than 8.96 million doses of the new coronavirus vaccine. Among them, the number of people vaccinated in the first dose reached 6.03 million, which means that the vaccination rate of permanent residents over 18 years old in Wuhan is 62%. |
| --- |

1、have you been vaccinated？

○Two shots have been vaccinated ○One shot has been vaccinated ○Haven’t

2、From May 1, 2021 to July 31, 2021, How do you think your frequency of travel to the following places has changed compared to 2019？

|  | Much Lower | Lower | Similar | Higher | Much higher |
| --- | --- | --- | --- | --- | --- |
| Restaurant |  |  |  |  |  |
| Outdoor Leisure Places |  |  |  |  |  |
| Indoor Leisure Places |  |  |  |  |  |
| Sport Places |  |  |  |  |  |
| Living Service Places |  |  |  |  |  |
| Shopping Places |  |  |  |  |  |

3、From May 1, 2021 to July 31, 2021, Are you willing to go to the following places？

|  | Almost None | Mild | Neutral | Quite | Strong |
| --- | --- | --- | --- | --- | --- |
| Restaurant |  |  |  |  |  |
| Outdoor Leisure Places |  |  |  |  |  |
| Indoor Leisure Places |  |  |  |  |  |
| Sport Places |  |  |  |  |  |
| Living Service Places |  |  |  |  |  |
| Shopping Places |  |  |  |  |  |

4、From May 1, 2021 to July 31, 2021, How necessity did you think it is to go to the following places？

|  | Almost None | Mild | Neutral | Quite | Strong |
| --- | --- | --- | --- | --- | --- |
| Restaurant |  |  |  |  |  |
| Outdoor Leisure Places |  |  |  |  |  |
| Indoor Leisure Places |  |  |  |  |  |
| Sport Places |  |  |  |  |  |
| Living Service Places |  |  |  |  |  |
| Shopping Places |  |  |  |  |  |

4.1From May 1, 2021 to July 31, 2021, Why did you think it is very necessary to go to this place？

|  | Totally disagree | Relatively disagree | Neutral | Relatively agree | Totally agree |
| --- | --- | --- | --- | --- | --- |
| For living |  |  |  |  |  |
| For social relations |  |  |  |  |  |
| For work |  |  |  |  |  |
| For mental health |  |  |  |  |  |

5、From May 1, 2021 to July 31, 2021, How risky do you think you are of contracting COVID-19？

|  | Almost None | Mild | Neutral | Quite | Strong |
| --- | --- | --- | --- | --- | --- |
| Restaurant |  |  |  |  |  |
| Outdoor Leisure Places |  |  |  |  |  |
| Indoor Leisure Places |  |  |  |  |  |
| Sport Places |  |  |  |  |  |
| Living Service Places |  |  |  |  |  |
| Shopping Places |  |  |  |  |  |

6、From May 1, 2021 to July 31, 2021, Are you concerned about the epidemic prevention policy related to the following places？

|  | Almost None | Mild | Neutral | Quite | Strong |
| --- | --- | --- | --- | --- | --- |
| Restaurant |  |  |  |  |  |
| Outdoor Leisure Places |  |  |  |  |  |
| Indoor Leisure Places |  |  |  |  |  |
| Sport Places |  |  |  |  |  |
| Living Service Places |  |  |  |  |  |
| Shopping Places |  |  |  |  |  |

7、From May 1, 2021 to July 31, 2021, Do you think the epidemic situation in the following places can be controlled through relevant epidemic prevention and control policies?？

|  | Almost None | Mild | Neutral | Quite | Strong |
| --- | --- | --- | --- | --- | --- |
| Restaurant |  |  |  |  |  |
| Outdoor Leisure Places |  |  |  |  |  |
| Indoor Leisure Places |  |  |  |  |  |
| Sport Places |  |  |  |  |  |
| Living Service Places |  |  |  |  |  |
| Shopping Places |  |  |  |  |  |

8、From May 1, 2021 to July 31, 2021, how many times a week did you go to the following places?

Restaurant times per week

Outdoor Leisure Places times per week

Indoor Leisure Places times per week

Sport Places times per week

Living Service Places times per week

Shopping Places times per week

**Supplementary text 2 Equation of minimum sample size for stratified random sampling**

$$n=\frac{P(1-P)}{\frac{e^{2}}{Z^{2}}+\frac{P(1-P)}{N}}$$

In which, P is the probability value, P=0.5,

e is the error value, e=5%,

Z is the confidence level, Z=90%

N is the total sample size of the study area, N=12,447,700

The minimum sample size n for this study is required to be 269 by calculation.

**Table S1 Demographic characteristics of respondents**

|  | N=450 | Percentage | Actual ratio ^a^ |
| --- | --- | --- | --- |
| **Gender** |  |  |  |
| Male | 224 | 49.70% | 50.80% |
| Female | 226 | 50.30% | 49.20% |
| **Age** |  |  |  |
| 18-24 | 76 | 16.80% | 6.83% |
| 25-30 | 68 | 15.10% | 8.68% |
| 31-34 | 70 | 15.50% | 8.67% |
| 35-39 | 73 | 15.20% | 8.71% |
| ≥40 | 163 | 36.20% | 29.07% |
| **Education** |  |  |  |
| High school and below | 68 | 15.11% | 52% ^b^ |
| College and above | 382 | 84.89% |  |
| **Household Monthly Income(yuan)** |  |  |  |
| ＜5000 | 76 | 16.89% | 4200 yuan ^c^ |
| 5000-10000 | 188 | 41.78% |  |
| ＞10000 | 186 | 41.33% |  |
| **Population** |  |  |  |
| 3 persons and below | 289 | 64.22% |  |
| 4persons and above | 161 | 35.78% |  |
| **Vaccine** |  |  |  |
| Two shots have been vaccinated | 397 | 88.20% | 77.63%^d^ |
| One shot has been vaccinated | 36 | 8.00% |  |
| Haven’t | 17 | 3.80% |  |

Note: a. Data from Wuhan Statistical Bureau, 2021. Hefei Statistical Yearbook.

http://tjj.wuhan.gov.cn/tjfw/tjnj/202112/t20211220_1877108.shtml

b. Undergraduate admission rate of Hubei Convince

c. Per capita monthly-income in average

d. Full immunization rate of people over 18 years old in Wuhan, as of 17:00 on July 20, 2021

**Table S2 Regression variable**

| Parameter | Variable definition |
| --- | --- |
| Willingness | Factor ‘Willingness’. 1=very low；2=low；3= middle；4=high；5=very high |
| Necessity | Factor ‘Necessity’. 1=very low；2=low；3= middle；4=high；5=very high |
| Risk | Factor ‘Risk’. 1=very low；2=low；3= middle；4=high；5=very high |
| Attention | Factor ‘Attention’. 1=very low；2=low；3= middle；4=high；5=very high |
| Controllability | Factor ‘Controllability’. 1=very low；2=low；3= middle；4=high；5=very high |
| Age | 1=18-24；2=25-30；3=31-34；4=35-49；5=≥40 |
| Gender | 1=Male；2=Female |
| Education | 1=High school and below；2=Bachelor degree and above |
| Income | 1=<5000，2=5000-10000，3=>10000 |
| Population | 1=3 persons and below，2=4 persons and above |
| Stage | 1= Before the epidemic，2= Before the community is blocked  3= After unblocking，4= One year after unblocking |

**Table S3 Spearman correlation analysis of perceptions in Restaurant**

Table S3-A Stage2

|  | Frequency | Willingness | Necessity | Risk | Attention | Controllabiliy |
| --- | --- | --- | --- | --- | --- | --- |
| Frequency | 1 | 0.439** | 0.426** | -0.238** | -0.155** | -0.110* |
| Willingness |  | 1 | 0.585** | -0.244** | -0.110* | -0.016 |
| Necessity |  |  | 1 | -0.293** | -0.136** | -0.052 |
| Risk |  |  |  | 1 | 0.535** | 0.318** |
| Attention |  |  |  |  | 1 | 0.376** |
| Controllability |  |  |  |  |  | 1 |

Table S3-B Stage3

|  | Frequency | Willingness | Necessity | Risk | Attention | Controllabiliy |
| --- | --- | --- | --- | --- | --- | --- |
| Frequency | 1 | 0.767** | 0.702** | -0.192** | 0.043 | 0.125** |
| Willingness |  | 1 | 0.801** | -0.289** | 0.113* | 0.164** |
| Necessity |  |  | 1 | -0.283** | 0.042 | 0.133** |
| Risk |  |  |  | 1 | 0.198** | 0.125** |
| Attention |  |  |  |  | 1 | 0.490** |
| Controllability |  |  |  |  |  | 1 |

Table S3-C Stage4

|  | Frequency | Willingness | Necessity | Risk | Attention | Controllabiliy |
| --- | --- | --- | --- | --- | --- | --- |
| Frequency | 1 | 0.676** | 0.569** | -0.210** | 0.174** | 0.149** |
| Willingness |  | 1 | 0.736** | -0.250** | 0.269** | 0.244** |
| Necessity |  |  | 1 | -0.261** | 0.198** | 0.205** |
| Risk |  |  |  | 1 | 0.047 | -0.170** |
| Attention |  |  |  |  | 1 | 0.471** |
| Controllability |  |  |  |  |  | 1 |

**Table S4 Spearman correlation analysis of perceptions in Outdoor Leisure places**

Table S4-A Stage2

|  | Frequency | Willingness | Necessity | Risk | Attention | Controllabiliy |
| --- | --- | --- | --- | --- | --- | --- |
| Frequency | 1 | 0.359** | 0.303** | -0.222** | -0.07 | -0.04 |
| Willingness |  | 1 | 0.664** | -0.285** | -0.091 | 0.018 |
| Necessity |  |  | 1 | -0.257** | -0.074 | 0.024 |
| Risk |  |  |  | 1 | 0.571** | 0.308** |
| Attention |  |  |  |  | 1 | 0.440** |
| Controllability |  |  |  |  |  | 1 |

Table S4-B Stage3

|  | Frequency | Willingness | Necessity | Risk | Attention | Controllabiliy |
| --- | --- | --- | --- | --- | --- | --- |
| Frequency | 1 | 0.779** | 0.710** | -0.345** | 0.026 | 0.119* |
| Willingness |  | 1 | 0.814** | -0.389** | 0.068 | 0.177** |
| Necessity |  |  | 1 | -0.409** | 0.014 | 0.142** |
| Risk |  |  |  | 1 | 0.253** | 0.023 |
| Attention |  |  |  |  | 1 | 0.421** |
| Controllability |  |  |  |  |  | 1 |

Table S4-C Stage4

|  | Frequency | Willingness | Necessity | Risk | Attention | Controllabiliy |
| --- | --- | --- | --- | --- | --- | --- |
| Frequency | 1 | 0.679** | 0.600** | -0.202** | 0.231** | 0.219** |
| Willingness |  | 1 | 0.754** | -0.234** | 0.282** | 0.266** |
| Necessity |  |  | 1 | -0.269** | 0.229** | 0.255** |
| Risk |  |  |  | 1 | 0.058 | -0.153** |
| Attention |  |  |  |  | 1 | 0.440** |
| Controllability |  |  |  |  |  | 1 |

**Table S5 Spearman correlation analysis of perceptions in Indoor Leisure places**

Table S5-A Stage2

|  | Frequency | Willingness | Necessity | Risk | Attention | Controllabiliy |
| --- | --- | --- | --- | --- | --- | --- |
| Frequency | 1 | 0.303** | 0.167** | -0.221** | -0.068 | -0.067 |
| Willingness |  | 1 | 0.434** | -0.123** | 0.006 | 0.041 |
| Necessity |  |  | 1 | -0.135** | -0.014 | 0.021 |
| Risk |  |  |  | 1 | 0.339** | 0.332** |
| Attention |  |  |  |  | 1 | 0.376** |
| Controllability |  |  |  |  |  | 1 |

Table S5-B Stage3

|  | Frequency | Willingness | Necessity | Risk | Attention | Controllabiliy |
| --- | --- | --- | --- | --- | --- | --- |
| Frequency | 1 | 0.776** | 0.698** | -0.178** | 0.082 | 0.141** |
| Willingness |  | 1 | 0.703** | -0.178** | 0.100* | 0.126** |
| Necessity |  |  | 1 | -0.227** | 0.099* | 0.115* |
| Risk |  |  |  | 1 | 0.124** | 0.014 |
| Attention |  |  |  |  | 1 | 0.417** |
| Controllability |  |  |  |  |  | 1 |

Table S5-C Stage4

|  | Frequency | Willingness | Necessity | Risk | Attention | Controllabiliy |
| --- | --- | --- | --- | --- | --- | --- |
| Frequency | 1 | 0.736** | 0.645** | -0.156** | 0.194** | 0.148** |
| Willingness |  | 1 | 0.734** | -0.113* | 0.238** | 0.196** |
| Necessity |  |  | 1 | -0.135** | 0.180** | 0.148** |
| Risk |  |  |  | 1 | 0.028 | -0.143** |
| Attention |  |  |  |  | 1 | 0.444** |
| Controllability |  |  |  |  |  | 1 |

**Table S6 Spearman correlation analysis of perceptions in Sport places**

Table S6-A Stage2

|  | Frequency | Willingness | Necessity | Risk | Attention | Controllabiliy |
| --- | --- | --- | --- | --- | --- | --- |
| Frequency | 1 | 0.411** | 0.354** | -0.182** | 0.09 | 0.049 |
| Willingness |  | 1 | 0.617** | -0.183** | 0.098* | 0.051 |
| Necessity |  |  | 1 | -0.134** | 0.095* | 0.071 |
| Risk |  |  |  | 1 | 0.362** | 0.339** |
| Attention |  |  |  |  | 1 | 0.429** |
| Controllability |  |  |  |  |  | 1 |

Table S6-B Stage3

|  | Frequency | Willingness | Necessity | Risk | Attention | Controllabiliy |
| --- | --- | --- | --- | --- | --- | --- |
| Frequency | 1 | 0.411** | 0.354** | -0.182** | 0.09 | 0.049 |
| Willingness |  | 1 | 0.617** | -0.183** | 0.098* | 0.051 |
| Necessity |  |  | 1 | -0.134** | 0.095* | 0.071 |
| Risk |  |  |  | 1 | 0.362** | 0.339** |
| Attention |  |  |  |  | 1 | 0.429** |
| Controllability |  |  |  |  |  | 1 |

Table S6-C Stage4

|  | Frequency | Willingness | Necessity | Risk | Attention | Controllabiliy |
| --- | --- | --- | --- | --- | --- | --- |
| Frequency | 1 | 0.832** | 0.746** | -0.095* | 0.324** | 0.196** |
| Willingness |  | 1 | 0.796** | -0.103* | 0.394** | 0.265** |
| Necessity |  |  | 1 | -0.123** | 0.345** | 0.231** |
| Risk |  |  |  | 1 | 0.051 | -0.178** |
| Attention |  |  |  |  | 1 | 0.430** |
| Controllability |  |  |  |  |  | 1 |

**Table S7 Spearman correlation analysis of perceptions in Living Service places**

Table S7-A Stage2

|  | Frequency | Willingness | Necessity | Risk | Attention | Controllabiliy |
| --- | --- | --- | --- | --- | --- | --- |
| Frequency | 1 | 0.309** | 0.349** | -0.196** | 0.038 | 0.033 |
| Willingness |  | 1 | 0.677** | -0.138** | 0.041 | 0.093* |
| Necessity |  |  | 1 | -0.185** | 0.018 | 0.051 |
| Risk |  |  |  | 1 | 0.472** | 0.308** |
| Attention |  |  |  |  | 1 | 0.552** |
| Controllability |  |  |  |  |  | 1 |

Table S7-B Stage3

|  | Frequency | Willingness | Necessity | Risk | Attention | Controllabiliy |
| --- | --- | --- | --- | --- | --- | --- |
| Frequency | 1 | 0.737** | 0.660** | -0.177** | 0.098* | 0.183** |
| Willingness |  | 1 | 0.779** | -0.189** | 0.165** | 0.227** |
| Necessity |  |  | 1 | -0.190** | 0.147** | 0.211** |
| Risk |  |  |  | 1 | 0.159** | -0.027 |
| Attention |  |  |  |  | 1 | 0.500** |
| Controllability |  |  |  |  |  | 1 |

Table S7-C Stage4

|  | Frequency | Willingness | Necessity | Risk | Attention | Controllabiliy |
| --- | --- | --- | --- | --- | --- | --- |
| Frequency | 1 | 0.692** | 0.614** | -0.036 | 0.282** | 0.235** |
| Willingness |  | 1 | 0.706** | -0.022 | 0.362** | 0.334** |
| Necessity |  |  | 1 | -0.028 | 0.265** | 0.260** |
| Risk |  |  |  | 1 | 0.046 | -0.132** |
| Attention |  |  |  |  | 1 | 0.525** |
| Controllability |  |  |  |  |  | 1 |

**Table S8 Spearman correlation analysis of perceptions in Shopping places**

Table S8-A Stage2

|  | Frequency | Willingness | Necessity | Risk | Attention | Controllabiliy |
| --- | --- | --- | --- | --- | --- | --- |
| Frequency | 1 | 0.335** | 0.337** | -0.078 | 0.086 | 0.088 |
| Willingness |  | 1 | 0.748** | -0.033 | 0.09 | 0.119* |
| Necessity |  |  | 1 | -0.082 | 0.087 | 0.094* |
| Risk |  |  |  | 1 | 0.566** | 0.278** |
| Attention |  |  |  |  | 1 | 0.473** |
| Controllability |  |  |  |  |  | 1 |

Table S8-B Stage3

|  | Frequency | Willingness | Necessity | Risk | Attention | Controllabiliy |
| --- | --- | --- | --- | --- | --- | --- |
| Frequency | 1 | 0.749** | 0.681** | -0.241** | 0.071 | 0.193** |
| Willingness |  | 1 | 0.818** | -0.236** | 0.144** | 0.228** |
| Necessity |  |  | 1 | -0.252** | 0.09 | 0.207** |
| Risk |  |  |  | 1 | 0.253** | 0.019 |
| Attention |  |  |  |  | 1 | 0.485** |
| Controllability |  |  |  |  |  | 1 |

Table S8-C Stage4

|  | Frequency | Willingness | Necessity | Risk | Attention | Controllabiliy |
| --- | --- | --- | --- | --- | --- | --- |
| Frequency | 1 | 0.634** | 0.562** | -0.125** | 0.187** | 0.226** |
| Willingness |  | 1 | 0.689** | -0.169** | 0.293** | 0.316** |
| Necessity |  |  | 1 | -0.167** | 0.197** | 0.300** |
| Risk |  |  |  | 1 | -0.002 | -0.243** |
| Attention |  |  |  |  | 1 | 0.500** |
| Controllability |  |  |  |  |  | 1 |

**Table S9 Confirmatory factor analysis and Goodness-of-fit statistics of Stage2**

| Reference | χ2/df | RMR | GFI | AGFI | IFI | TLI | CFI | RMSEA |
| --- | --- | --- | --- | --- | --- | --- | --- | --- |
| standard | <5.00 | <0.04 | >0.90 | >0.90 | >0.90 | >0.90 | >0.90 | <0.05 |
| Restaurant | 0.48 | 0.01 | 1.00 | 0.99 | 1.00 | 1.01 | 1.00 | 0.00 |
| Outdoor Leisure | 0.98 | 0.01 | 1.00 | 0.99 | 1.00 | 1.00 | 1.00 | 0.00 |
| Indoor Leisure | 1.36 | 0.01 | 1.00 | 0.98 | 1.00 | 0.99 | 1.00 | 0.03 |
| Sport | 0.27 | 0.00 | 1.00 | 1.00 | 1.00 | 1.02 | 1.00 | 0.00 |
| Living Service | 2.43 | 0.01 | 1.00 | 0.97 | 1.00 | 0.98 | 1.00 | 0.06 |
| Shopping | 1.83 | 0.01 | 1.00 | 0.98 | 1.00 | 0.99 | 1.00 | 0.04 |

Note: χ2/df = Normal Theory Weighted Least Squares Chi-Square/Degrees of Freedom; RMR = Root Mean Square Residual; GFI = Goodness of Fit Index; AGFI = Adjusted Goodness of Fit Index; IFI = Incremental Fit Index; TLI = Tucker-Lewis Coefficient; CFI = Comparative Fit Index; RMSEA = Root Mean Square Error of Approximation

**Table S10 Confirmatory factor analysis and Goodness-of-fit statistics of Stage3**

| Reference | χ2/df | RMR | GFI | AGFI | IFI | TLI | CFI | RMSEA |
| --- | --- | --- | --- | --- | --- | --- | --- | --- |
| standard | <5.00 | <0.04 | >0.90 | >0.90 | >0.90 | >0.90 | >0.90 | <0.05 |
| Restaurant | 1.05 | 0.01 | 1.00 | 0.99 | 1.00 | 1.00 | 1.00 | 0.01 |
| Outdoor Leisure | 2.09 | 0.01 | 1.00 | 0.97 | 1.00 | 0.99 | 1.00 | 0.05 |
| Indoor Leisure | 1.11 | 0.01 | 1.00 | 0.99 | 1.00 | 1.00 | 1.00 | 0.02 |
| Sport | 0.76 | 0.01 | 1.00 | 0.99 | 1.00 | 1.00 | 1.00 | 0.00 |
| Living Service | 1.89 | 0.01 | 1.00 | 0.98 | 1.00 | 0.99 | 1.00 | 0.05 |
| Shopping | 0.89 | 0.01 | 1.00 | 0.99 | 1.00 | 1.00 | 1.00 | 0.00 |

**Table S11 Confirmatory factor analysis and Goodness-of-fit statistics of Stage4**

| Reference | χ2/df | RMR | GFI | AGFI | IFI | TLI | CFI | RMSEA |
| --- | --- | --- | --- | --- | --- | --- | --- | --- |
| standard | <5.00 | <0.04 | >0.90 | >0.90 | >0.90 | >0.90 | >0.90 | <0.05 |
| Restaurant | 0.95 | 0.01 | 1.00 | 0.99 | 1.00 | 1.00 | 1.00 | 0.00 |
| Outdoor Leisure | 0.73 | 0.01 | 1.00 | 0.99 | 1.00 | 1.01 | 1.00 | 0.00 |
| Indoor Leisure | 1.93 | 0.01 | 1.00 | 0.97 | 1.00 | 0.98 | 1.00 | 0.05 |
| Sport | 1.49 | 0.01 | 1.00 | 0.98 | 1.00 | 0.99 | 1.00 | 0.03 |
| Living Service | 5.00 | 0.01 | 1.00 | 0.93 | 0.99 | 0.92 | 0.99 | 0.10 |
| Shopping | 0.86 | 0.00 | 1.00 | 0.99 | 1.00 | 1.00 | 1.00 | 0.00 |

**Table S12 Path analysis of perceived risk, necessity, and willingness**

Table 12-1 Stage2

| Stage2 | Restaurant | Outdoor Leisure | Indoor Leisure | Sport | Living Service | Shopping |
| --- | --- | --- | --- | --- | --- | --- |
| Risk←Attention | 0.434*** | 0.524*** | 0.177*** | 0.207*** | 0.405*** | 0.574*** |
| Risk←Controllability | 0.161*** | 0.063 | 0.173*** | 0.186*** | 0.061 | 0.010 |
| Necessity←Controllability | 0.045 | 0.055 | 0.024 | 0.042 | 0.049 | 0.056 |
| Necessity←Attention | 0.023 | 0.044 | 0.005 | 0.063 | 0.066 | 0.162** |
| Necessity←Risk | -0.082* | -0.165*** | -0.065** | -0.080** | -0.135*** | -0.080 |
| Willingness←Risk | -0.021 | -0.098*** | -0.040* | -0.081*** | -0.030 | 0.020 |
| Willingness←Necessity | 0.486*** | 0.636*** | 0.383*** | 0.561*** | 0.690*** | 0.729*** |
| Willingness←Attention | 0.000 | 0.024 | 0.012 | 0.041 | 0.032 | 0.012 |

Table 12-2 Stage3

| Stage3 | Restaurant | Outdoor Leisure | Indoor Leisure | Sport | Living Service | Shopping |
| --- | --- | --- | --- | --- | --- | --- |
| Risk←Attention | 0.187*** | 0.320*** | 0.111*** | 0.062 | 0.219*** | 0.399*** |
| Risk←Controllability | 0.040 | -0.113* | -0.041 | -0.024 | -0.146** | -0.154** |
| Necessity←Controllability | 0.173** | 0.138** | 0.055 | 0.028 | 0.160** | 0.193*** |
| Necessity←Attention | 0.026 | 0.078 | 0.057 | 0.188*** | 0.097** | 0.088 |
| Necessity←Risk | -0.281*** | -0.415*** | -0.176*** | -0.299*** | -0.190*** | -0.243*** |
| Willingness←Risk | -0.095** | -0.096** | -0.018 | -0.062** | -0.049 | -0.053 |
| Willingness←Necessity | 0.789*** | 0.806*** | 0.659*** | 0.740*** | 0.731*** | 0.788*** |
| Willingness←Attention | 0.105*** | 0.092 | 0.020 | 0.075*** | 0.057* | 0.105** |

Table 12-3 Stage4

| Stage4 | Restaurant | Outdoor Leisure | Indoor Leisure | Sport | Living Service | Shopping |
| --- | --- | --- | --- | --- | --- | --- |
| Risk←Attention | 0.182** | 0.168** | 0.100* | 0.129** | 0.141** | 0.180** |
| Risk←Controllability | -0.333*** | -0.297*** | -0.215*** | -0.272*** | -0.248*** | -0.427*** |
| Necessity←Controllability | 0.102 | 0.171** | 0.061 | 0.084 | 0.185** | 0.271*** |
| Necessity←Attention | 0.179*** | 0.192*** | 0.127** | 0.285*** | 0.157*** | 0.080 |
| Necessity←Risk | -0.243*** | -0.257*** | -0.124** | -0.133** | -0.028 | -0.147*** |
| Willingness←Risk | -0.068* | -0.046 | -0.012 | -0.013 | 0.002 | -0.045 |
| Willingness←Necessity | 0.708*** | 0.750*** | 0.766*** | 0.800*** | 0.675*** | 0.633**** |
| Willingness←Attention | 0.147*** | 0.134*** | 0.097*** | 0.131*** | 0.173*** | 0.163*** |

Note: a. ***p<0.001, **p<0.01, *p<0.05

b. CMIN/DF<5.000, RMR<0.040, GFI>0.900, AGFI>0.900, IFI>0.900, CFI>0.900, TLI>0.900, TLI>0.900, RMSEA<0.050. More details in Table S2-S4

**Figure S1 Existing Cases in Wuhan**


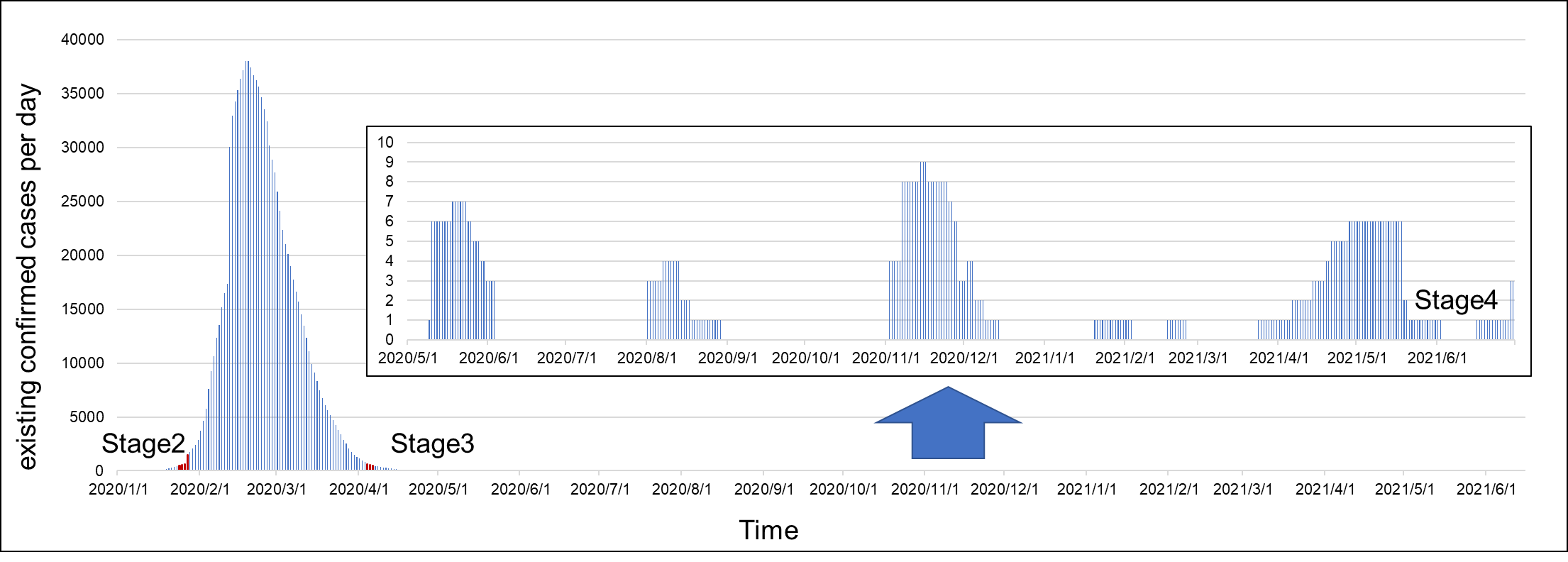

Supplement: Supplementary file 1 — Supplementary Information. [file 41598_2023_32009_MOESM1_ESM.docx]
